# Clinical infectious outcomes associated with biofilm-related bacterial infections: a retrospective chart review

**DOI:** 10.1186/s12879-015-0972-2

**Published:** 2015-06-07

**Authors:** Alice E. Barsoumian, Katrin Mende, Carlos J. Sanchez, Miriam L. Beckius, Joseph C. Wenke, Clinton K. Murray, Kevin S. Akers

**Affiliations:** Infectious Disease Service, San Antonio Military Medical Center, JBSA Fort Sam Houston, San Antonio, TX USA; Infectious Disease Clinical Research Program, Uniformed University of the Health Sciences, Bethesda, MD USA; United States Army Institute of Surgical Research, JBSA Fort Sam Houston, San Antonio, TX USA; Department of Clinical Investigation, San Antonio Military Medical Center, JBSA Fort Sam Houston, San Antonio, TX USA

**Keywords:** Biofilm, Chronic infections, Risk factors, Trauma-related infections, Burn

## Abstract

**Background:**

Biofilms are associated with persistent infection. Reports characterizing clinical infectious outcomes and patient risk factors for colonization or infection with biofilm forming isolates are scarce. Our institution recently published a study examining the biofilm forming ability of 205 randomly selected clinical isolates. This present study aims to identify potential risk factors associated with these isolates and assess clinical infectious outcomes.

**Methods:**

221 clinical isolates collected from 2005 to 2012 and previously characterized for biofilm formation were studied. Clinical information from the associated patients, including demographics, comorbidities, antibiotic usage, laboratory values, and clinical infectious outcomes, was determined retrospectively through chart review. Duplicate isolates and non-clinical isolates were excluded from analysis. Associations with biofilm forming isolates were determined by univariate analysis and multivariate analysis.

**Results:**

187 isolates in 144 patients were identified for analysis; 113 were biofilm producers and 74 were not biofilm producers. Patients were primarily male (78 %) military members (61 %) with combat trauma (52 %). On multivariate analysis, the presence of methicillin-resistant *Staphylococcus aureus* (*p* < 0.01, OR 5.09, 95 % CI 1.12, 23.1) and *Pseudomonas aeruginosa* (*p* = 0.02, OR 3.73, 95 % CI 1.46, 9.53) were the only characteristics more likely to be present in the biofilm producing isolate group. Infectious outcomes of patients with non-biofilm forming isolates, including cure, relapse/reinfection, and chronic infection, were similar to infectious outcomes of patients with biofilm-forming isolates. Mortality with initial infection was higher in the biofilm producing isolate group (16 % vs 5 %, *p* = 0.01) but attributable mortality was low (1 of 14). No characteristics examined in this study were found to be associated with relapse/reinfection or chronic infection on multivariate analysis.

**Conclusions:**

Bacteria species, but not clinical characteristics, were associated with biofilm formation on multivariate analysis. Biofilm forming isolates and non-biofilm forming isolates had similar infectious outcomes in this study.

## Background

The ability of microorganisms to form biofilms, a sessile mode of growth intrinsically associated with antimicrobial resistance and recalcitrant infection, is a current topic of interest as it may constitute a virulence factor in human infections [1]. This phenotype has been observed *in vivo* through scanning electron microscopy in many situations. This observation has led to the implication of biofilms in a majority of human infections [2]. Biofilm formation also has been linked with poor wound healing [3], burn wound ulceration [4], and medical implanted device related infections [5]. Treatment of infections with biofilm forming bacteria is notoriously difficult, and requires higher doses or combination of antibiotics, and removal of foreign bodies when implicated in device related infections [1, 6].

Despite an increased understanding of this process and the observation of the presence of biofilms in important infections, most studies have described this phenomenon *in vitro.* There are few studies which establish a link between the biofilm-forming phenotype of these organisms and clinical infectious outcomes or that identify risk factors for expression of the phenotype. Limitations with the study of biofilms *in vitro* include the omission of host factors present at sites of infection (e.g. plasma proteins), which can stimulate biofilm formation [7], the lack of standardization among laboratory methods for characterizing biofilm phenotypes *in vitro*, and the lack of clinically available *in vivo* assays. Recently, we examined the phenotypic and genotypic characteristics of 205 randomly selected clinical bacterial isolates with respect to their ability to form biofilms [8], and we noticed biofilm formation was observed in isolates serially recovered from presumed ongoing infections in several patients. Clinical information associated with the isolates, however, was not collected, and the significance of this observation was not known. To better understand clinical risk factors for acquisition of biofilm-forming organisms and then to examine whether an isolate’s ability to form biofilms played a role in the outcomes of infection, we retrospectively gathered clinical information from patients contributing these isolates.

## Methods

### Bacterial isolates

Two hundred twenty one clinical isolates of the species *Acinetobacter calcoaceticus-baumannii* complex, *Escherichia coli*, *Klebsiella pneumoniae*, *Pseudomonas aeruginosa*, and *Staphylococcus aureus*, collected from January 2005 to June 2012 were randomly selected from a bacterial repository at San Antonio Military Medical Center (Fort Sam Houston, TX, USA), including the 205 previously described [8]. This strain repository includes all isolates recovered from patients enrolled in prospective studies at our institution, regardless of their antimicrobial susceptibility, as well as multidrug resistant isolates recovered from the general patient population. The biofilm forming ability of these isolates was previously characterized *in vitro* using the semi-quantitative microtiter plate biofilm formation assay, in which the absorbance of solubilized crystal violet of each selected isolate is compared to a known biofilm forming strain [9]. In this way, biofilm formation was graded as positive or negative as compared to a positive control.

Study subjects contributing to the previously reported isolates [8], subject to additional exclusion criteria, were selected for further study. Duplicate isolates, defined as isolates of the same species and PFGE type, recovered from the same anatomical site within 10 days of the matching isolate and having concordant categorical biofilm-forming phenotypes, were omitted. Thus, only single-patient unique isolates were included in the study. Non-clinical isolates, such as those related to surveillance swabs obtained for infection control purposes, were also excluded, along with isolates recovered from patients <18 years old and those which could not be linked to source patients.

### Clinical data

For the study subjects with bacterial isolates meeting the defined criteria and included in this study, the potential clinical predictors of infection or colonization and associated clinical outcomes of infection were extracted from the medical record. These included basic demographics, medical comorbidities (coronary artery disease, chronic kidney disease, diabetes), circumstances of injury (military service, deployment exposure, combat trauma), presence of burn and percent total body surface area burned (TBSA). Laboratory data including hematocrit, white blood cell count, platelet count, erythrocyte sedimentation rate (ESR), and c-reactive protein (CRP) on the day of admission, day of isolate recovery, and day of discharge were recorded. Microbiological data included the identity of primary and co-infecting organisms, as well as receipt of antibiotics prior to recovery of clinical isolates. The anatomic source of isolates and their association with clinical support devices (such as implanted orthopedic devices, intravascular catheters, urinary catheters, etc.) was also obtained from the medical record, using radiographs where appropriate.

Infecting organisms were distinguished from colonizing organisms by chart review based upon clinical circumstances, using established definitions (asymptomatic bacteriuria [10], central line associated bloodstream infection [11], catheter associated urinary tract infection [12], ventilator associated pneumonia [13]), and reviewer physician judgment. Outcomes including clinical cure, relapse/reinfection and chronic infection were also recorded. Length of follow up was greater than 24 months. Relapse or reinfection was defined as recovery of an organism from the same site more than 10 days after a period of culture negativity, or clinical quiescence after treatment with presumed cure. Chronic infection was defined as the continued, intermittent recovery of organisms while on antibiotics, or immediate return of infectious symptoms after cessation of antibiotic therapy, with no period of clinical quiescence while off of antibiotic therapy. In-hospital death was recorded, and the documented cause of death was cross- referenced to autopsy information, where available.

All data were collected through chart review by a single investigator to minimize variation in collection strategy. Study design was approved by the San Antonio Military Medical Center Institutional Review Board with a waiver of informed consent.

### Statistical analysis

Data were examined for normal vs. non-parametric distribution using the Kolmogorov-Smirnov test. For univariate analysis, differences in frequencies were determined using Pearson’s chi-square or Fisher’s exact tests, while the Mann–Whitney-U test was used to examine differences in the medians of continuous data. Standard multivariate analysis was performed using multiple logistic regression, including in the model predictive variables having univariate p-values <0.2. Statistical calculations were performed using SPSS (IBM SPSS v19.0) and p-values of <0.05 were considered statistically significant.

## Results

### Patient demographics and characteristics

Of the 221 isolates, 34 isolates from 26 patients were excluded. Of these, 18 were non-clinical isolates, 13 isolates from 5 patients were duplicates, two were obtained from patients <18 years old, and one could not be unequivocally associated to a source patient. Therefore, 187 isolates associated with unique infectious events in 144 patients were included in the analysis. Both biofilm producing and non-biofilm producing bacteria were recovered from 12 patients. Of those, 7 patients had complex polymicrobial wounds with the isolates recovered from the same site; the other events were unrelated. No similarities were noted amongst the patients. Patients were primarily male (78 %) military members (61 %) with combat trauma (52 %). Demographic information of the patients from whom biofilm producing bacteria were recovered (*n* = 79) was not significantly different from demographic information of the patients from whom non-biofilm producing bacteria were recovered (*n* = 53) (Table 1). Including these 12 patients in the analysis did not reveal any significant differences in demographic variables between the two groups (data not shown).

**Table 1:** Demographics of patients with biofilm producing isolates versus patients with non-biofilm producing isolates^a^

	Biofilm producing isolate group	Non-biofilm producing isolate group	P-value
Number of Patients	79	53	
Median Age, years (IQR)	30 (22, 44)	34 (24, 60)	0.64
Male	66 (84 %)	37 (70 %)	0.10
Military Service	53 (67 %)	28 (53 %)	0.13
Combat Trauma	44 (56 %)	24 (45 %)	0.30
Burn Patients	30 (38 %)	12 (23 %)	0.08
Median % TBSA burn (IQR)	52 % (36, 78)	55 % (48, 66)	0.31
Coronary Artery Disease	4 (5 %)	6 (11 %)	0.16
Chronic Kidney Disease	1 (1 %)	1 (2 %)	0.33
Diabetes mellitus	5 (6 %)	8 (15 %)	0.16

TBSA- total body surface area

^a^excluding 12 patients represented in both groups

### Analysis of possible associations with biofilm formation

In univariate analysis, male gender, military service and combat trauma were significantly more common in the biofilm producing isolate group; whereas the only clinical complication associated with the non-biofilm producing group among this cohort was diabetes. Biofilm producing isolates were more often associated with and isolated from various anatomical sites, including respiratory cultures, wound cultures, as well as orthopedic device related infections. In contrast, non-biofilm producing isolates were significantly associated with urine cultures. Bacterial species significantly observed to be associated with the biofilm producing group were *P. aeruginosa* and methicillin-resistant *S. aureus* (MRSA); whereas *E. coli* was more commonly associated with the non-biofilm producing group. Interestingly, no significant correlation for either group was determined for isolates of *A. calcoaceticus-baumannii* complex and *K. pneumoniae* (Table 2).

**Table 2:** Clinical and microbiologic characteristics associated with isolates of the biofilm producing and non-biofilm producing groups

	Biofilm producing isolates	Non-biofilm producing isolates	Univariate P-Value	Multivariate P-Value
Number of Isolates	113	74		
Male	101 (89 %)	56 (76 %)	0.01	NS
Military	86 (76 %)	41 (55 %)	<0.01	NS
Combat Trauma	74 (65 %)	35 (47 %)	0.01	NS
Burn	54 (48 %)	27 (36 %)	0.13	NS
Median % TBSA burn (IQR)	60 % (37, 79)	55 % (48, 63)	0.36	
Coronary Artery Disease	4 (4 %)	7 (9 %)	0.09	NS
Chronic Kidney Disease	1 (1 %)	1 (1 %)	0.76	
ESRD	0 (0 %)	2 (3 %)	0.08	NS
Diabetes	6 (5 %)	11 (15 %)	0.03	NS
Median Hct on Culture Date g/dl (IQR)	24.2 (22, 30)	24.1 (22, 31)	0.67	
Median WBC on Culture Date 10^3^ cells/μl (IQR)	9.6 (6, 13)	9 (6, 12)	0.62	
Median ESR on Culture Date mm/hr (IQR)	95 (57, 114)	79 (67, 106)	0.77	
Median CRP on Culture Date mg/dl (IQR)	16.6 (8, 24)	12.25 (5, 20)	0.21	
Received Appropriate Empiric Antibiotics	47 (42 %)	32 (43 %)	0.74	
Median Prior Duration of Antibiotics, Days (IQR)	4 (2, 8)	4 (2, 8)	0.52	
*A. calcoaceticus-baumannii* complex (*n* = 53)	29 (55 %)	24 (45 %)	0.32	
*E. coli* (*n* = 34)	5 (14 %)	29 (85 %)	<0.001	0.01
*K. pneumoniae* (*n* = 44)	32 (71 %)	12 (27 %)	0.06	NS
*P. aeruginosa* (*n* = 34)	28 (82 %)	6 (18 %)	<0.01	<0.01
*S. aureus* (*n* = 22)	19 (86 %)	3 (14 %)	0.01	NS
MSSA (*n* = 6)	5 (83 %)	1 (17 %)	0.24	
MRSA (*n* =16)	14 (87.5 %)	2 (12.5 %)	0.02	0.02
Polymicrobial Event^a^	39 (35 %)	20 (27 %)	0.28	
Blood Culture	20 (18 %)	21 (28 %)	0.08	NS
Central Line Associated	2 (10 %, *n* = 20)	4 (19 %, *n* = 21)	0.09	NS
Respiratory Culture	10 (9 %)	1 (1 %)	0.03	NS
Urine Culture	3 (3 %)	18 (24 %)	<0.01	NS
Foley Catheter Associated	1 (33 %, *n* = 3)	4 (22 %, *n* = 18)	0.50	
Wound Culture	80 (71 %)	34 (46 %)	<0.01	NS
Orthopedic Device Related	15 (19 %, *n* = 80)	4 (12 %, *n* = 34)	<0.01	NS
Median Number of Surgeries (IQR)	5.5 (2, 9)	6 (2, 10)	0.56	

*TBSA* total body surface area, *Hct* hematocrit, *WBC* white blood cell count, *ESR* erythrocyte sedimentation rate, *CRP* c-reactive protein, *MSSA* methicillin susceptible *S. aureus*, *MRSA* methicillin-resistant *S. aureus*

^a^isolate recovered from a site with more than one organism recovered

Laboratory data, medical comorbidities, and antibiotic exposure prior to recovery of isolate were not different between the two groups. In multivariate analysis, presence of MRSA (*p* < 0.01) (OR 5.09, 95 % CI 1.12, 23.1) and *P. aeruginosa* (*p* = 0.02) (OR 3.73, 95 % CI 1.46, 9.53) were the only characteristics found to be more likely to be present in the biofilm producing isolate group with *E. coli* less likely present in that group (*p* = 0.01), with an odds ratio of 0.07 and 95 % CI (0.03, 0.2).

### Clinical infectious outcomes of patients with respect to biofilm formation

Infectious outcomes of patients with biofilm producing isolates including infection, infection versus colonization, cure of first infection, relapse/reinfection, and chronic infection, were similar to those with non-biofilm producing isolates. All-cause mortality during the initial infection was significantly more common in patients carrying biofilm producing isolates (16 % vs 5 %, *p* = 0.01) (Table 3). Of the three patients who died during initial infection with a non-biofilm forming isolate, one died due to sepsis and multisystem organ failure caused by a study isolate; the remaining two deaths were attributed to a fungal brain abscess and one to a necrotic bowel, respectively. Two of these patients had suffered burns, 2 were male, with a median age 36, and a median TBSA burn of 55 %. The isolates were *A. calcoaceticus-baumannii* complex (*n* = 2), and *P. aeruginosa* (*n* = 1), and were recovered blood (*n* = 2), or wound cultures (*n* = 1).

**Table 3:** Correlation of clinical outcomes of infections to biofilm producing versus non-biofilm producing isolates

	Biofilm producing isolates	Non-biofilm producing isolates	P-value
Total Infecting Isolates	88 (78 %, *n* = 113)	60 (81 %, *n* =74)	0.60
Death during Initial Infection	14 (16 %, *n* = 88)	3 (5 %, *n* = 60)	0.01
Cure Without Relapse/Reinfection^a^	55 (74 %, *n* = 74)	46 (81 %, *n* = 57)	0.39
Relapse/Reinfection^a^	18 (24 %, *n* = 74)	11 (19 %, *n* =57)	0.49
Chronic Infection^a^	1 (1 %, *n* = 74)	0	1

^a^excluding patients who died during initial infection

Of the 14 patients who died during the first infection with a biofilm-forming isolate, only one was due to multisystem organ failure from sepsis attributable to a study isolate. The remaining 13 deaths were due to ischemic bowel/abdominal compartment syndrome (*n* = 5), sepsis unrelated to study isolate (*n* = 3), withdrawal of care due to underlying non-infectious illness (*n* = 2), invasive fungal infection (*n* = 1), ventilator associated pneumonia/respiratory failure (*n* = 1), and multisystem organ failure (*n* = 1). These patients also suffered burn injuries (*n* = 10) with a median TBSA of 55 %, and were male (*n* = 12), with a median age of 32. The isolates from those patients included *A. calcoaceticus-baumannii* complex (*n* = 3), *K. pneumoniae* (*n* = 3), *P. aeruginosa* (*n* = 7), and MRSA (*n* = 1). Ten of these study isolates were recovered from wounds, 2 from blood culture, and 1 from a sputum culture.

### Analysis of characteristics associated with relapse/reinfection or chronic infection

Characteristics associated with relapse/reinfection or chronic infection identified on univariate analysis included higher percentage TBSA (77 % versus 53.5 %, *p* <0.01), infection with *P. aeruginosa* (23 % vs 9 %, *p* = 0.03) or infection with methicillin-sensitive *S. aureus* (MSSA) (17 % vs 1 %, *p* < 0.01). Recovery of an isolate from a wound culture was more common in patients who were cured (68 % vs 37 %, *p* <0.01). Laboratory data, medical comorbidities, antibiotic exposure prior to recovery, and association with medical devices was implicated on univariate analysis with relapse/reinfection or chronic infection. In multivariate analysis, no factors were significantly associated with relapse/reinfection or chronic infection (Table 4).

**Table 4:** Clinical and microbiological characteristics associated with patients experiencing cure, without relapse/reinfection, or chronic infection^a^

	Total cure, initial infection (*n* = 101)	Relapse/reinfection, or chronic infection (*n* = 30)	P-value	Multivariate P-value
Median Age, years (IQR)	28 (23, 41)	30 (22, 41)	0.44	
Male	91 (91 %)	24 (80 %)	0.14	NS
Military Service	75 (75 %)	17 (57 %)	0.06	NS
Combat Trauma	68 (68 %)	16 (53 %)	0.16	NS
Burn	38 (38 %)	14 (47 %)	0.37	
% TBSA burn (IQR)	53.5 % (28, 60)	77 % (59, 85)	<0.01	NS
Coronary Artery Disease	3 (3 %)	0	0.34	
Chronic Kidney Disease	0	0		
ESRD	0	0		
Diabetes	6 (6 %)	5 (17 %)	0.06	NS
Median Hct on Culture Date (IQR)	24.2 (22, 30)	23.6 (22, 27)	0.53	
Median WBC on Culture Date (IQR)	9.7 (7, 13)	7 (5, 12)	0.11	NS
Median ESR on Culture Date (IQR)	91.5 (65, 114)	106 (104, 114)	0.48	
Median CRP on Culture Date (IQR)	18.9 (7, 21)	16.1 (9, 30)	0.14	NS
Received Appropriate Empiric Antibiotics	39 (39 %)	16 (53 %)	0.28	
Median Prior Duration of Antibiotics, Days (IQR)	6 (2, 8)	4 (2, 9)	0.57	
*A. calcoaceticus-baumannii* complex	34 (34 %)	6 (20 %)	0.41	
*E. coli*	19 (19 %)	5 (17 %)	0.79	
*K. pneumoniae*	25 (25 %)	7 (23 %)	0.87	
*P. aeruginosa*	9 (9 %)	7 (23 %)	0.03	NS
*S. aureus*	14 (14 %)	6 (20 %)	0.41	
MSSA	1 (1 %)	5 (17 %)	<0.01	NS
MRSA	12 (13 %)	1 (3 %)	0.14	NS
Polymicrobial Event	41 (41 %)	7 (23 %)	0.09	NS
Blood Culture	23 (23 %)	11 (37 %)	0.13	NS
Central Line Associated	9 (39 % *n* = 23)	0	0.12	NS
Respiratory Culture	4 (4 %)	4 (13 %)	0.08	NS
Urine Culture	6 (6 %)	4 (13 %)	0.24	
Foley Catheter Associated	2	0	0.44	
Wound Culture	68 (68 %)	11 (37 %)	<0.01	NS
Orthopedic Device Related	13 (19 %, *n* = 68)	5 (25 %, *n* = 11)	0.50	
Median Number of Surgeries (IQR)	7 (4, 9)	3 (2, 9)	0.15	NS

^a^Patients who died during initial infection are excluded from this analysis

## Discussion

Notably, no clinical variables were found to be independently associated with biofilm formation in our study. Unlike previous studies, we did not observe biofilm formation to be statistically associated with relapse/reinfection, or chronic infections [14]. These prior studies examine mainly chronic diabetic or vascular stasis ulcers, which were not the type of wounds in our population; many of our patients underwent aggressive serial debridement of wounds, which is a unique aspect of trauma care. This result also differs from our earlier publication on these isolates, in which biofilm formation was observed in isolates serially recovered from ongoing infections in several patients [8]. With the present study, clinical information recovered from chart review was able to differentiate colonizing from infecting organisms, distinguish between discrete infections, and assess for outcome. A case control study recently published from our institution, with a different patient population and bacterial isolates, demonstrated that biofilm formation was associated with persistence of skin and soft tissue wound infection beyond 14 days [3]. Although no differences were found between biofilm producing isolates versus non-producing isolates and cure, correlation to wound persistence or duration of infection was not examined in this study.

Interestingly, we did not find significant differences in previous antibiotic exposure or duration of antibiotic exposure prior to organism recovery among biofilm formers either on univariate or multivariate analysis. Some authors have found that antibiotic exposure can induce biofilm formation *in vitro* [15]. It is possible that this phenomenon is not replicated *in vivo*, or that the analysis is confounded by the relatively high rate of previous antibiotic exposure in this population in whom severe trauma is common.

More deaths occurred with initial infections by biofilm-producing isolates overall, but the attributable mortality (i.e. death from infection) remained low (1 out of 14). The reasons for this are not immediately clear as both groups were similar with respect to presence of burn, TBSA of burn, gender, age, and site of recovery; although low numbers in the comparator group make drawing conclusions difficult. Our literature search did not reveal any studies addressing this issue.

In univariate analysis, higher percentage TBSA was found to be associated with relapse/reinfection, or chronic infection. This is similar to findings indicating that the risk of burn wound infection increases in proportion to the amount of body surface area burned [16]. MSSA was more likely to be associated with relapse/reinfection, and chronic infection, but this analysis is limited by the small sample size (6 isolates). Study isolates recovered from wound infections were more likely to be cured without relapse or reinfection; perhaps this reflects the ability to obtain a surgical cure through tissue debridement. However, no clinical variables or microbiological characteristics were found in this study in multivariate analysis to be independent predictors of relapse/reinfection or chronic infection.

There are several limitations notable in this study. As a retrospective study, our data are subject to selection bias and misclassification bias, particularly since we were not able to control for these potential influences. Utilization of a randomly selected sample had consequences in microorganism representation as well as demographics. Species were unevenly represented in this sample (6 MSSA versus 53 *A. calcoaceticus-baumannii* complex). However, this distribution was similar to prospectively-acquired data from military casualties having persistent wound infections [3]. A majority of the isolates selected were biofilm producers. This may be due to the selection of isolates from a repository containing many multidrug resistant isolates. This composition may not accurately represent the frequency of biofilm formation among isolates in an unselected population, but did allow for the study of large numbers of biofilm and non-biofilm forming isolates.

Common medical comorbidities, such as coronary artery disease (10 of 144 patients) and diabetes (13 of 144 patients) were also underrepresented; this may have been the result of sampling bias from our burn center, the young age of patients in the cohort, or inaccurate documentation of patients’ past medical history. There are likely too few cases to reasonably draw conclusions regarding the presence of biofilm forming isolates and these medical comorbidities.

With regards to medical device-related infections, despite a large overall isolate database, we observed only 19 orthopedic device related infections, 5 urinary catheter related infections, and 6 central line related infections. The small numbers in these cohorts could explain why no association was found with catheter associated infections and also explain why the association with orthopaedic devices was not significant on multivariate analysis. Future studies could include a sample size determined by power analysis to detect differences for all potential associations.

Unlike previous studies [17], recovery of organism from a polymicrobial culture site was not correlated with biofilm producing isolates. Data examining the biofilm producing capability of the co-located organisms were not available due to the retrospective nature of this study; it is possible that a non-study isolate was a biofilm producer and co-located with the study isolate. With these in mind, future studies should investigate the characteristics of each isolate in a clinical polymicrobial culture to better understand the nature of interactions, and multiple organisms commonly isolated together from polymicrobial wound infections could be studied together to determine the influence of multiple species on biofilm formation.

Ultimately, the *in vitro* formation of biofilms may not be related to the *in vivo* phenotype, and the *in vitro* formation of biofilms may underrepresent the biofilm forming capability of an organism. For example, plasma enhances the *in vitro* biofilm formation of *S. aureus*. Current techniques assessing *in vitro* biofilm formation often exclude factors that impact expression of this phenotype in an *in vivo* infection, and laboratory protocols standardizing biofilm assessment are lacking. It is also possible that while isolates may have the ability to form biofilms *in vitro* and *in vivo*, this phenotype may not always be expressed in a particular infection. Furthermore, the link between the *in vitro* phenotype and clinical outcome is still unclear, and further studies are required to establish this link.

## Conclusions

Bacterial species, but not clinical variables, were found to be independently associated with biofilm formation in the present study. No clinical or microbiological factors were found to be significantly associated with relapse/reinfection, or chronic infection on multivariate analysis. This analysis of potential clinical risk factors and infectious outcomes of a large data set is an important addition to the understanding of this phenotype’s role in disease. Continued investigation into infectious outcomes is warranted to balance our *in vitro* understanding with the *in vivo* implications*.*
